# Live Bacterial Prophylactics in Modern Poultry

**DOI:** 10.3389/fvets.2020.592312

**Published:** 2020-10-28

**Authors:** Graham A. J. Redweik, Jared Jochum, Melha Mellata

**Affiliations:** ^1^Department of Food Science and Human Nutrition, Iowa State University, Ames, IA, United States; ^2^Interdepartmental Microbiology Graduate Program, Iowa State University, Ames, IA, United States

**Keywords:** probiotics, live vaccines, food safety, immunology, chickens

## Abstract

Commercial poultry farms frequently use live bacterial prophylactics like vaccines and probiotics to prevent bacterial infections. Due to the emergence of antibiotic-resistant bacteria in poultry animals, a closer examination into the health benefits and limitations of commercial, live prophylactics as an alternative to antibiotics is urgently needed. In this review, we summarize the peer-reviewed literature of several commercial live bacterial vaccines and probiotics. Per our estimation, there is a paucity of peer-reviewed published research regarding these products, making repeatability, product-comparison, and understanding biological mechanisms difficult. Furthermore, we briefly-outline significant issues such as probiotic-label accuracy, lack of commercially available live bacterial vaccines for major poultry-related bacteria such as *Campylobacter* and *Clostridium perfringens*, as well research gaps (i.e., probiotic-mediated vaccine adjuvancy, gut-brain-microbiota axis). Increased emphasis on these areas would open several avenues for research, ranging from improving protection against bacterial pathogens to using these prophylactics to modulate animal behavior.

## Introduction

Poultry animals like layers and broilers are some of the most critical food animals, with 90 billion tons of chickens meat being produced globally per year (1) and 290 eggs consumed per capita in the United States (2). Over the years, poultry have been domesticated to maximize particular functions like meat and egg production. Although selecting for greater weight gain and egg-laying rates has improved poultry productivity, specific selection for bacterial diseases resistance has not been pursued as diligently. This is problematic, as poultry animals are becoming increasingly at-risk for bacterial infections given the push for cage-free rearing [reviewed by (3)] and serve as major reservoirs for foodborne pathogens like *Salmonella* and *Campylobacter*, contaminating their products [(4); reviewed in (5)]. Furthermore, the emergence of antimicrobial-resistant (AMR) bacterial pathogens threaten poultry animals and humans health alike (6). Specifically, avian pathogenic *Escherichia coli* (APEC), *Pasteurella multocida*, and *Mycoplasma gallisepticum* are causal agents of disease and mortality in poultry animals, which have the potential to harbor AMR genes [(7–9); reviewed in (10)]. Additionally, chickens are common carriers of bacteria like *Salmonella* and *Campylobacter*, which reside as commensals in their gastrointestinal tract [reviewed in (11, 12)]. However, these bacteria are frequent contaminators of poultry products and cause gastrointestinal disease in human consumers [reviewed in (13–15)]. Even worse, these microbes can horizontally-exchange AMR genes with commensals or other pathogens (16–18). This has created a dangerous situation in which bacterial pathogens (chicken and human alike) may become highly-difficult to treat with conventional antibiotics. Thus, cost-effective additives that can boost resistance to pathogenic and AMR bacteria are needed to further optimize both poultry health and productivity.

Among the strategies currently used to promote productivity in animal agriculture includes use of live microorganisms. This includes live bacterial vaccines, which are attenuated bacteria typically used to immunize animals against particular pathogens [reviewed in (19)], and probiotics, which are live, non-attenuated microbes that confer health benefits to the animal host (20). Probiotics are typically delivered via feed, although spray and intraocular administration are commonly-used to deliver live bacterial vaccines. Additionally, both live bacterial vaccines and probiotics can be cultured *in vitro*, which drastically lowers production costs [reviewed in (21); reviewed in (22)]. In this review, we will outline live bacterial vaccines and probiotics commercially-available in poultry, describing the peer-reviewed studies using these commercial products in poultry animals. Additionally, we discuss probiotic labels and reliability-concerns. Lastly, this review will discuss the potential for novel live vaccines, synergism between live prophylactics, and a possible role for live prophylactics in less-studied biological mechanisms such as behavior.

## Live Bacterial Vaccines

### General

The earliest recorded live bacterial vaccination was in 1884, where Spanish clinicians utilized a weak *Vibrio cholerae* isolate to combat cholera outbreaks (23). Techniques for purposefully-attenuating bacterial strains while maintaining immunogenicity have been improved, using targeted modifications at the genetic level (24). Concerns over virulence-reversion by live bacterial vaccines have driven researchers to develop antigen-based vaccines incapable of sustaining disease. However, the successful development of antigen vaccines with long-term efficacy is relatively rare, mainly due to evolutionary adaptations by pathogens (i.e., antigenic loss/drift, serotype diversity) and design, as antigens have much-fewer epitopes compared to live bacteria vaccines, limiting protection against multiple, antigenically-diverse strains of a certain pathogen (25). Thus, live bacterial vaccines provide a lucrative alternative that can circumvent many issues with vaccination in poultry.

Like their wild-type counterparts, live bacterial vaccines can be easily cultured *in vitro* with low input costs, providing an inexpensive means of manufacturing large quantities of vaccine vs. the protein extraction steps required for antigen-based vaccines (21). Therefore, these vaccines can simultaneously prevent disease caused by their wild-type parent bacterium as well as additional pathogens (bacterial, viral, etc.) because cross-reactivity or via genetic insertions of genes encoding foreign antigen (24), creating an avenue for broad protection unachievable by many prophylactics currently available. In this review, we summarize characteristics and peer-reviewed findings for commercial live *Salmonella enterica, Escherichia coli, Mycoplasma gallisepticum*, and *Pasteurella multocida* vaccines available for poultry in Table 1.

**Table 1:** Summary of live bacterial vaccines commercially available for poultry application.

**Commercial live bacterial vaccine**	**Company**	**Species (strain), additional attenuations**	**Peer-reviewed findings**
AviPro® Megan® Vac 1	Elanco	*Salmonella* Typhimurium, Δ*cya*Δ*crp*	Decreased *Salmonella* invasion and intestinal colonization (26) Vaccine efficacy may be inconsistent and recoverable from internal organs (27) Decrease *Salmonella* detection in commercial carcasses (28)
Gallivac® SE	Merial select	*Salmonella* Enteritidis, Δ*ade*Δ*his*	Induces IgA and IgY in intestinal washes; Reduced Typhimurium and Enteritidis in ceca and internal organs (29) Reduced intestinal macrophages and CD4+ cells but increased CD8+ cell recruitment (30) Reduced *S*. Heidelberg in ceca when challenged at 21 but not 3 days post-hatch (31)
Poulvac® ST	Zoetis	*Salmonella* Typhimurium, Δ*serC*Δ*aroA*	Reduced *S*. Typhimurium load in liver and ceca (32) Increased inflammatory gene expression in splenic cells (33)
Poulvac® *E. coli*	Zoetis	*Escherichia coli*, O78 serotype, Δ*a roA*	Reduced O78 APEC bacterial load in internal organs (34) Did not protect chickens against O1 APEC; spray administration had superior protection against O78 APEC challenge vs. drinking water (35)
MG TS-11	Merial select	*Mycoplasma gallisepticum* (TS-11 strain)	Protection against R-strain *M. gallisepticum* challenge without change in productivity (36, 37)
MYCOVAC-L®	Merck	*Mycoplasma gallisepticum* (Intervet 6/85 strain)	Improved vaccine viability in PBS vs. distilled water (38) Protective immunity against *M. gallisepticum*, vaccination at recommended-dose may reduce egg production (39)
Poulvac® MycoF	Zoetis	*Mycoplasma gallisepticum* (F strain)	Protection against *M. gallisepticum-*induced airsacculitis (40) Intraocular vaccination induces greatest immune response (41)
AviPro® MG-F	Elanco	*Mycoplasma gallisepticum* (F strain)	Protection against M. gallisepticum-induced airsacculitis (40) Lless antibody production vs. MycoF (42) induced superior immune responses vs. TS-11 and MYCOVAC-L® (43, 44)
M-NINEVAX®-C	Merck	*Pasteurella multocida* (M-9 strain)	Potent antibody response against *P. multocida* (45)
PM-ONEVAX®-C	Merck	*Pasteurella multocida* (PM-1 strain)	Protection against *P. multocida* and high antibody titer (45)

## Commercial Live Bacterial Vaccines

### Salmonella

Although *Salmonella enterica* induces inflammatory in the chicken gut at an early age (46–48), this bacterium can persist by restructuring the intestinal environment to promote immunological tolerance, allowing for asymptomatically-shedding via feces from poultry animals [reviewed in (11)], resulting in potential contamination of meat and egg products. Human consumption of these contaminated poultry products is one of the major routes of salmonellosis incidence in the United States (49, 50). Live *Salmonella* vaccines are typically delivered orally via spray or drinking water to reduce *Salmonella* load in poultry. To improve food safety, live *Salmonella* vaccines are augmented with genetic deficiencies to limit intestinal replication while maintaining high levels of immunogenicity (24), although serotype and genetic attenuations are important drivers of vaccine efficacy (51, 52). Furthermore, these vaccines can be readily-modified to carry exogenous antigens (53, 54), enabling protection against additional pathogens.

Megan® Vac-1 is a Δ*cya*Δ*crp S*. Typhimurium vaccine [parent strain Δ3761 or UK-1 (55)], genetically-attenuated to knockout adenylate cyclase (Δ*cya*) and cAMP receptor protein (Δ*crp*). These mutations reduce pathogenicity and persistence of this live vaccine in the intestine while maintaining high immunogenicity, as demonstrated by the decrease of a challenge *Salmonella* invasion and intestinal colonization in vaccinated layer pullets (26). However, the protection of this vaccine against *Salmonella* appears to be inconsistent. A previous study testing protection against a wild-type *S*. Typhimurium strain in broiler chicks found the Megan® Vac-1 only reduced challenge *Salmonella* load in one of the two challenge experiments, although the failure in the first experiment may have been related to *in ovo* antibiotic administration (27). Furthermore, the vaccine strain was frequently-recovered from internal organs and ceca of vaccinated birds (27), although sampled animals were only 1-week-old and thus are not representative of broilers at final slaughter. In support of this, Dórea and colleagues determined that Megan® Vac-1 significantly-reduced detection of *Salmonella* in commercial broiler carcasses, minimizing carcass condemnation (28).

Poulvac® ST (Zoetis) is another metabolically-attenuated *S*. Typhimurium strain with Δ*serC* (phosphoserine aminotransferase) and Δ*aroA* (3-phosphoshikimate 1-carboxyvinyltransferase) deletions. Despite these deletions, Poulvac® ST is still immunogenic, inducing anti-lipopolysaccharide IgA and IgY responses in intestinal washes at day 13 (29) despite a reduction in ileal macrophages and CD4^+^ T cells (30). Furthermore, vaccinated broilers had reduced *S*. Heidelberg loads in the ceca when challenged at 21 days old (31). This response may have been facilitated by recruitment of intestinal CD8^+^ T cells (30), which have been previously demonstrated to improve *Salmonella* clearance in chickens (56). However, this vaccine was unable to reduce *Salmonella* Heidelberg load in the ceca when challenged at 3 days (31). This may be due to serovar-specific, as Bailey and colleagues found that Poulvac® ST alone did reduce challenge *Salmonella* Typhimurium and Enteritidis invasion of internal organs and ceca colonization in 3 and 13-day-old chicks (29).

Unlike the previously-described *Salmonella* vaccines, Gallivac® SE (Merial Select) is a *S*. Enteritidis strain (Δ*ade*Δ*his*) developed via chemical mutagenesis. Similar to the other vaccines, Gallivac® SE can provide protection against non-Enteritidis serovars, as orally-delivered Gallivac® SE reduced *S*. Typhimurium burden in the liver and ceca up to week 71 in layer hens vs. unvaccinated hens (32). Although live *Salmonella* vaccines are normally given orally, intraocular administration of Gallivac® SE increased IFNΔ, IL-8, and iNOs production by splenic cells (33), suggesting that this vaccine is capable of inducing robust immune responses, which extend from mucosal barriers. Unfortunately, to the authors' knowledge, these are the only two peer-reviewed studies which investigated the immunological potential of Gallivac® SE *in vivo*.

#### Escherichia coli

One of the major drivers of mortality and carcass condemnation in poultry, APEC are a major problem in commercial production (57). In addition, APEC are characterized by the possession of large virulence plasmids that often carry numerous resistances to antibiotics and heavy metals [reviewed in (58–60)]. These plasmids can be horizontally-transferred to other gut commensals as well bacteria like *Salmonella* (61), making the reduction of APEC in poultry a major priority. Given their antigenic variability (62), vaccines with broad protection have proved problematic. Notably, APEC colonize the gastrointestinal tract as commensals (63, 64) and only cause colibacillosis when they translocate the lung epithelium upon fecal aerosolization (65, 66). Thus, orally-delivered live vaccines are a feasible strategy to reduce abundances of these microbes in the gut while also inducing systemic immunity for extraintestinal resistance.

As of this review, Poulvac® *E. coli* (Zoetis) is the only live *E. coli* vaccine for poultry on the market. Poulvac® *E. coli* has a O78 serotype and, similar to Poulvac® ST, is a Δ*aroA* mutant. When implemented in broilers, this vaccine increased the number of healthy carcasses and reduced collibacillosis of a O78 APEC field isolate compared to non-vaccinated controls (67). Similarly, Poulvac® *E. coli* decreased bacterial load of an O78 APEC in internal organs compared to non-vaccinated controls, possibly related to improvements in *E.coli* O78 antigen-specific IgY serum levels and splenocyte proliferation (34). However, this protection appears to be serotype-specific, as Poulvac® *E. coli* did not confer any protection against challenge with an O1 APEC serotype (35). Furthermore, route appears to be a major determinant of efficacy, as Poulvac® *E. coli* administered to broilers via drinking water did not confer any protection to an O78 APEC, whereas coarse spray-administration did (35).

#### Mycoplasma gallisepticum

The etiological agent of chronic respiratory disease and infectious sinusitis in poultry animals [reviewed in (68); reviewed in (69)], *M. gallisepticum* is a major cause of carcass condemnation, reductions in egg-laying efficiency and weight gain, and mortality in commercial poultry (70–73). Given its antigen variability, details regarding its entire pathogenesis are unclear (74). Initially, *M. gallisepticum* binds to sialic acid residues on lung epithelial cells (75) and can cause damage high inflammatory damage locally (76) or in deeper lymphoid tissues like the bursa of Fabricius (77). Although birds at all ages are susceptible to this bacterium, immunocompromised birds are especially at-risk for infection (78). Currently, live vaccines are typically used to prevent *M. gallisepticum* infection in poultry.

MG TS-11 (Merial Select) is a live attenuated strain of *M. gallisepticum* that is delivered via intraocular route (i.e., eyedrop). Its complete genome sequence is available to the public (79). This vaccine strain can prevent development of clinical airsacculitis, peribronchitis, and interstitial pneumonia via R-strain *M. gallisepticum* challenge without reducing egg-laying productivity (36, 37). More recently, research groups have sought to improve the efficacy of the TS-11 vaccine. Muneta and colleagues found that a recombinant TS-11 expressing IFNΔ increased cellular immunity via increased splenocyte-IFNΔ production and a non-edematous infiltration of heterophils into the trachea mucosa (36). Furthermore, TS-304, a TS-11 derivative that expresses the cytadherence molecule GapA, was shown to be more efficacious than TS-11 at a lower dose (80) likely related to its ability to more-effectively improve tracheal barrier function (76).

MYCOVAC-L® (Merck) is an attenuated 6/85 strain of *M. gallisepticum*. Similar to TS-11, its complete genome sequence is readily-available (38). Typically delivered via spray, rehydration of the vaccine via distilled water (standard practice for many bacterial vaccines) results in much lower MYCOVAC-L® viability vs. resuspension in PBS (38). In addition, although vaccination dose at the manufacturer's recommendation confers protective immunity against virulent *M. gallisepticum*, egg production may be negatively-impacted. However, hens previously vaccinated with fifteen times the recommended dose did not exhibit any deficiencies in egg-laying efficiency and produced more antibodies (39), suggesting a greater inoculum concentration is needed to negate certain side effects of MYCOVAC-L®.

Poulvac® MycoF (Zoetis) is an F strain of *M. gallisepticum* typically administered via spray. Using spray, Evans and colleagues showed MycoF-vaccinated animals did not exhibit *M. gallisepticum-*induced airsacculitis compared to control animals (40). Similar to MYCOVAC-L®, resuspension medium prior to MycoF immunization had a major impact on viability and antibody production immediately post-vaccination (81). When given via intraocular route, MycoF demonstrated protection against spread of *M. gallisepticum* in a co-mingled poultry system (82), suggesting that different vaccination routes may deliver similar success. However, when delivered in the same study via eyedrop, nares, or orally, intraocular MycoF vaccination induced the greatest antibody response (41), although this study did not investigate differences in *M. gallisepticum* resistance *in vivo*.

Another F strain vaccine, AviPro® MG-F (Elanco) was similarly able prevent airsacculitis via *M. gallisepticum* challenge (40). Although recommended delivery is in drinking water, Evans and colleagues found that when delivered via spray, MG-F delivered similar protection against *M. gallisepticum* infection as MycoF. Additionally, MG-F induced less antibody production vs. MycoF at one and ten-times recommended dose (42). However, MG-F induced superior immune responses compared to TS-11 and MYCOVAC-L® live vaccines (43, 44), suggesting that these *M. gallisepticum* vaccines induce immune responses in a vaccine strain-specific manner.

#### Pasteurella multocida

In the 1880s, Louis Pasteur developed one of the earliest live bacterial vaccines by isolating avian *P. multocida*, the etiological agent of fowl cholera, and using old cultures for immunization (83). Although a commensal member of the oropharyngeal microbiota, *P. multocida* can become an opportunistic pathogen in the respiratory tract (84). If able to bypass the lung epithelium, it can induce a highly-lethal septicemia (i.e., fowl cholera), causing major economic losses in poultry production (85, 86), though turkeys are more-affected (85). Thus, wing-web immunization of live *P. multocida* vaccines, superior to bacterin-based vaccines for this pathogen (87), is the most common method of prophylaxes against this pathogen. Although the exact mechanisms for protection are somewhat unclear, these live vaccines can induce broad protection independent of serotype and lipopolysaccharide composition (88). However, to the author's knowledge, very little peer-reviewed research has been performed using these *Pasteurella* live vaccines.

M-NINEVAX®-C (Merck) is an M-9 vaccine strain used in vaccinating commercial turkey flock against *P. multocida* and in combination with other live vaccines (89). Of the few studies using this vaccine, Sharaf and colleagues found this vaccine induced a potent antibody response against *P. multocida* (45). Similarly, PM-ONEVAX®-C, a PM-1 strain, induces protection against *P. multocida* challenge *in vivo*, accompanied with a high antibody titer (90). Unfortunately, no peer-reviewed studies on these live vaccines have been performed in the last two decades.

## Probiotics

### General

Probiotics are live microorganisms including bacteria (i.e., *Lactobacillus acidophilus*) and yeast (i.e., *Saccharomyces cerevisiae*) that are commonly supplemented in poultry feed to improve animal well-being through a variety of mechanisms. Probiotics have a variety of functions in host, which are mainly triggered by their outer membrane composition and metabolic outputs. In this section, we will discuss major classes of probiotics used in poultry and their general functions. Furthermore, we will summarize the findings of peer-reviewed studies using commercial probiotic products, organized by probiotics composed of a single class or mixture of classes (Table 2). This review will be limited to the effects of these commercial products on host immune function, productivity measures, and bacterial resistance (specifically, intestinal colonizers like *Salmonella, Campylobacter, E. coli*, and *Clostridium perfringens*). Given the limited focus on mechanisms with these commercial probiotics in poultry, this review will outline observed outcomes in-general in peer-reviewed studies.

**Table 2:** Summary of commercial probiotics available for poultry application.

**Probiotic class**	**Commercial product**	**Microbial taxa (per label)**	**Peer-reviewed findings**
LAB	FloraMax®-B11	*Lactobacillus salivarius, Pediococcus parvulus*	Immunomodulation and reduced intestinal NFκB transcription (91) Reduced colonization of *S*. Enteritidis and improved barrier function (92) Reduced *S*. Enteritidis, *E. coli*, and *C. jejuni in vitro* (93) Improved gut morphology and decreased *Salmonella* (94) Increased weight gain, reduced *Clostridium perfringens* and necrotic enteritis-induced mortality (95) Sequencing methods yield different taxonomic identifications (93)
	Cylactin®	*Enterococcus faecium* NCIMB 1045	Improved body weight, reduced colonization of *Clostridium* spp. and *E. coli* in excreta and intestine; increased levels of lactate, short-chain and branched-chain fatty acids (96) No change in intestinal *S*. Enteritidis load (97)
*Bacillus subtilis*	GalliPro®	*Bacillus subtilis* DSM 17229	Improved performance and decreased ammonia emission (98) Reduced *Salmonella* colonization (99) Increased body weight, feed conversion, and crude protein liberation (100) Complete elimination of *Clostridium perfringens* colonization in ileum (101)
	CloSTAT®	*Bacillus subtilis* PB6	Increased body weight, feed intake; no change in ileal lactobacilli nor *Clostridium perfringens* (102) Reduced mortality against *E. coli* (103) Reduced *Clostridium perfringens* in ileum (102)
	Norum™	*Bacillus amyloliquefaciens* AM0938*Bacillus amyloliquefaciens* JD17*Bacillus subtilis* AM1002	*In vitro* reduction of *Salmonella, E. coli, Clostridium difficile* (104) Reduced gut leakage (105–107) Decreased necrotic enteritis lesions (105) Lower horizontal transfer (108) and liver translocation (104) of *E. coli*
Mixture	Lavipan®	*Lactobacillus casei* LOCK 0915, *L. lactis* IBB 500, *Carnobacterium divergens* S-1, *L. plantarum* LOCK 0862, *Saccharomyces cerevisiae* LOCK 0141	Limited colonization of *Campylobacter* and *Salmonella* Enteritidis (109) Improved villi width and surface area in duodenum, jejunum, and ileum (110) Reduced *Clostridium* and *E. coli* (96)
	PrimaLac®	*Lactobacillus acidophilus, L. casei, Enterococcus faecium, Bifidobacterium bifidum*	Limited colonization of *Salmonella* and *E. coli* (111)*, Campylobacter jejuni* (112), *Clostridium perfringens* (144) No changes in ceca lactobacilli (113, 115) Age-dependent alterations in immune gene expression via *in ovo* (116)
	MicroGuard®	*Bacillus licheniformis, B. megaterium, B. mesentricus, B. polymyxa, B. subtilis, Saccharomyces boulardii, Bifidobacterium bifidum, Lactobacillus acidophilus, L. bulgaricus, L. plantarum, Streptococcus faecium*	Improved broiler performance; reduced *Salmonella* Enteritidis and *E. coli* (117)
	Gro-2-Max®	*Lactobacillus acidophilus, Pediococcus pentosaceus, P. acidilactici, Bacillus subtilis, Saccharomyces cerevisiae*	Increase in intestinal Enterobacteriaceae (118, 119) although this finding is inconsistent (119) Adjuvant activity against *Salmonella* and APEC using live *Salmonella* vaccine (118) Reduced total triglycerides, low-density lipoprotein cholesterol; altered circulatory immune parameters (119) Label inaccuracy (*Saccharomyces pastorianus* vs. *S. cerevisiae*) (118)

### Lactic Acid Bacteria

*Lactobacillus, Enterococcus*, and *Pediococcus* are gut commensals and examples of lactic acid (i.e., lactate in the ionized form)-producing bacteria (LAB), which protect against pathogens by several mechanisms. LAB are frequently used by poultry producers in part due their ability to produce several digestive enzymes (amylases, chitinases, lipases, phytases, and proteases), which greatly enhance the digestive process and improve feed conversion [reviewed in (120)]. Lactate is the major product of sugar metabolism across all LAB (121). Lactate can inhibit pathogenic bacterial growth by lowering the pH of the intestinal environment (122) or directly by disturbing normal bacterial metabolism (123). Select LAB also produce inhibitory compounds like bacteriocins, which are bactericidal compounds that target specific microorganisms (124). LAB can also directly stimulate immune cells via secretory factors (125) and toll-like receptor stimulation (125, 126). Given this wide array of functions, LAB are common components in several commercial probiotics used in poultry agriculture. Some examples of commercial LAB products for poultry animals are discussed below.

FloraMax®-B11 is a probiotic supplement composed of *Lactobacillus salivarius* and *Pediococcus parvulus*. Upon oral challenge with *Salmonella* Enteritidis, broilers fed FloraMax®-B11 showed reduced colonization of *Salmonella* Enteritidis, improved gut barrier function, and reduced percentages of heterophils, lymphocytes, eosinophils, and basophils of peripheral blood compared to control broilers (92). Given the role of immune inflammation in clearing intestinal *Salmonella* (127) and observed-reduction of circulatory immune cells, this suggests this product may have directly-reduced *Salmonella* load in the intestine. This mechanism of direct competition is supported in which each probiotic bacterium in FloraMax®-B11 directly-reduced *Salmonella* Enteritidis, *E. coli*, and *Campylobacter jejuni* growth *in vitro* (93). Although Prado-Rebolledo and colleagues did not investigate phenotypic changes in these immune cells, FloraMax®-B11 reduces intestinal gene expression associated with the NFκB complex and aldose reductase (91), suggesting this probiotic also reduces expression of inflammatory genes. In combination with the perinatal supplement EarlyBird (Pacific Vet Group USA Inc.), FloraMax®-B11 was shown to improve gut morphology and significantly decrease *Salmonella* recovery, incidence, and horizontal transmission to broiler chicks (94). Lastly, broilers supplemented with FloraMax®-B11 showed significant body weight gain, lower total *Clostridium perfringens* (the causal agent of necrotic enteritis), and lower necrotic enteritis-induced mortality when compared to control broilers after *C. perfringens* challenge (95).

Cylactin® is composed of a single LAB, *Enterococcus faecium* NCIMB 1045 (128). Implementation of Cylactin® to the diets of broilers has shown to have positive effects on average body weight, greatly-decreased counts of *Clostridium* spp. and *E. coli* in intestinal tract and excreta compared to controls, and had improved lactate production as well as short-chain and branched-chain fatty acids (96). However, Cylactin® alone did not reduce *Salmonella* Enteritidis load in the layer intestine (97). Although tested in a non-avian model, administration of Cylactin® in the diet of piglets showed significantly reduced mucus-adherent extraintestinal pathogenic strains of *E. coli* (129), suggesting that this probiotic could have direct effects on APEC found in the chicken intestine.

### Bacillus

Similarly to LAB, *Bacillus* species secrete digestive enzymes that improve feed conversion and competitive exclusion, which limit the ability of pathogens to invade the host (130–132). However, *B. subtilis* specifically limits pathogen colonization by production and secretion of lipopeptides and other antimicrobial compounds, as 4–5% of a *B. subtilis* genome is devoted to the production of antimicrobials [reviewed in (133)]. In contrast to LAB, *B. subtilis* can form endospores (134), improving their survival in the harsh conditions of the intestinal tract and food preparation processes better than other probiotics (135, 136). *B. subtilis* has also been shown to alter the morphology of the intestinal tract via elevated villi height and increased villi height-to-crypt depths (137), increasing the surface area for nutrient absorption. Notably, the host immune response toward *B. subtilis* is driven based on whether it is in its metabolically-inactive (i.e., endospore) or active (i.e., vegetative) state, as T cell differentiation was driven toward inflammatory, intracellular T_H_1 responses and extracellular T_H_2 responses via sporous and vegetative *B. subtilis*, respectively (138). Thus, de-sporulation in the intestine is a critical factor that could have major consequences on the host immune response.

GalliPro® consists of a single strain, *B. subtilis* DSM 17229 which improved performance, and reduced ammonia emission from the excreta in broilers (98). GalliPro® has been shown to reverse loss of splenic mass in *Salmonella*-infected birds, although no immune parameters were changed when non-infected birds were fed this probiotic (67). Furthermore, GalliPro® increased the liberation of crude protein from the diet, consequently decreasing broiler feeding costs and increasing body weight and feed conversion ratios (100). However, this study did not show whether GalliPro® was directly involved in this liberation or indirectly through a shift in the microbiota. Addition of GalliPro® to feed reduced *Salmonella* in cecum samples and greatly reduced *Salmonella*-positive drag swabs when compared to control broilers (99). Lastly, GalliPro® facilitates complete elimination of *C. perfringens* colonization in the ileum of challenged birds (101).

CloSTAT® contains a single strain of *B. subtilis*, PB6. When included to the diet of *C. perfringens*-challenged broilers at 1 × 10^9^ CFU CloSTAT®/g feed, these broilers had statistically increased body weight and feed intake counts compared to challenged broilers without probiotics (139). However, CloSTAT® supplementation did not significantly change bacterial load of lactobacilli nor *C. perfringens* in the ileal digesta (139).When investigating the mortality rates from *E. coli* challenge comparing broilers fed CloSTAT®, control, and antibiotic growth promoters, CloSTAT® showed reduction comparable to the antibiotic growth promoter (both significantly compared to control) (103). Similarly to GalliPro®, CloSTAT® also reduced *C. perfringens* colonization of the ileum upon challenge (102).

Norum™ is a direct-fed microbial culture that consists of two *B. amyloliquefaciens* strains (AM0938 and JD17) Addition of Norum™ has shown an increase in productivity parameters like body weight, body weight gain and feed conversion (105, 140). Norum™ greatly reduced the gut permeability and leakage of mucosal, immunological effectors like IgA into serum (105). In a necrotic enteritis model in which birds were challenged by *Salmonella* Typhimurium*, Eimeria maxima*, and *Clostridium perfringens* at days 1, 13, and 18–19 post-hatch, respectively, Norum™ significantly improved lesion scores (105). Lastly, *in ovo* administration of Norum™ to the feed greatly decreased the horizontal transmission of virulent *E. coli* and infection of broiler chickens during hatch, possibly through alterations of microbiota composition and community structure (108).

### *Bifidobacterium, Saccharomyces*, and Multi-Species Probiotics

To the authors' knowledge, there are no commercial poultry probiotics solely-constituted of *Bifidobacterium* spp. However, *Bifidobacterium* spp. are widely used in combination with *Lactobacillus* probiotics (ex: PrimaLac®) and other combination products (ex: MicroGuard®). *Bifidobacterium* directly affects IgA secretion in the gut (141) as well as stimulates professional phagocytes and pancreatic elastase production via secretion of the serine protease inhibitor Serpin (142). This pro-inflammatory mechanism action suggests that *Bifidobacterium* Serpin-production is involved in the homeostasis of the gut microbiota. Additionally, *Bifidobacterium* spp. produces acetate and lactate, which are subsequently-used by microbial gut fermenters to produce butyrate and propionate (143). These two short-chain fatty acids (SCFAs) promote colonic regulatory T cell differentiation (144, 145) as well as increase bactericidal functions of intestinal macrophages (146). Furthermore, the high GC content of the *Bifidobacterium* genome interacts with TLR9 that is present on the surface of mammalian immune cells (141, 147), although it is not clear whether *Bifidobacterium* DNA has a similar effect on the avian analog TLR21 (148).

Although the scope of this review is live bacterial prophylactics, the eukaryotic *Sacchormyces* species *S. cerevisiae* and *S. boulardii* [although *S. boulardii* is arguably a sub-species of *S. cerevisiae* (149)] are widely-implemented in poultry probiotic mixtures (i.e., Gro-2-Max® and MicroGuard®, respectively) and thus will be briefly-mentioned. Despite these two species being highly-similar, *S. boulardii* has greater heat and acid tolerance vs. *S. cerevisiae*, making it more competitive in the gut microenvironment [reviewed in (150)]. Additionally, both *Saccharomyces* species increased SCFA production via shifts in the microbiome (151, 152). Furthermore, *S. cerevisiae* and *S. boulardii* can directly-eliminate pathogens via secretory antimicrobials (153, 154). However, only *S. boulardii* appears to possess membrane-associated inulin, which can agglutinate pathogens (155, 156).

Lavipan® consists of several LAB (*Lactobacillus casei* LOCK 0915, *Lactobacillus lactis* IBB 500, *Carnobacterium divergens* S-1, and *Lactobacillus plantarum* LOCK 0862, all at 1 × 10^9^ CFU/g product) and *Saccharomyces cerevisiae* LOCK 0141 (1 × 10^7^ CFU/g) and was shown to competitively exclude pathogenic bacteria such as *Campylobacter* spp. and *Salmonella* Enteritidis (109). This probiotic also improved villi morphometric parameters (i.e., villus width and surface area) of the duodenum, jejunum, and ileum compared to control group (110). Lavipan® supplementation also caused reduced *Clostridium* spp. and *Escherichia coli* when compared to the control broilers, which was increased with the addition of prebiotics (i.e., raffinose family oligosaccharides) (96), which are non-viable food components like that improve host health via direct modification of the commensal microbiota (157). Thus, adding prebiotics to commercial probiotic products may improve health outcomes in poultry animals.

PrimaLac® is composed of *Lactobacillus acidophilus, Lactobacillus casei, Enterococcus faecium*, and *Bifidobacterium bifidium*, all at 1 × 10^6^ CFU/g (158). The use of PrimaLac has been shown to limit the colonization of *Salmonella* and *E. coli* (111) as well as *C. jejuni* (115). However, this probiotic does not induce any changes in ceca lactobacilli (113, 115). Supplementation of this probiotic to broilers *in ovo* produced an upregulation of *iNOS*, crucial for improving macrophage-killing of bacteria, in the ileum at day-of-hatch. However, later time points observed PrimaLac®-mediated downregulation of immune genes encoding toll-like receptors, cytokines, and iNOS in the ileum and ceca tonsil (116). The addition of PrimaLac® to the feed of turkey poults reduced *Salmonella* colonization upon challenge when compared to the control birds (111). When compared to an antibiotic growth promoter and control groups, addition of PrimaLac® increased reduction of *C. perfringens*, as well as improved broiler performance (114).

MicroGuard® contains 11 microorganisms (*Bacillus licheniformis, B. megaterium, B. mesentricus, B. polymyxa, B. subtilis, Saccharomyces boulardii, Bifidobacterium bifidum, Lactobacillus acidophilus, L. bulgaricus, L. plantarum*, and *Streptococcus faecium*) (159). The addition of MicroGuard® to the commercial broilers increased final bodyweight, weight gain, high density lipoprotein, triglyceride, and antibody titers against Newcastle disease and avian influenza levels (117). The addition of MicroGuard® also limited colonization of both *Salmonella* Enteritids and *E. coli* due to the above mentioned mechanisms, competitive exclusion, and possibly the production of bacteriocins (117).

Lastly, Gro-2-Max® is a multi-species probiotic product containing LAB (*Lactobacillus acidophilus, Pediococcus pentosaceus, P. acidilactici*), *Bacillus subtilis*, and *Saccharomyces cerevisiae*. When comparing route and length of treatment, Gro-2-Max® supplementation via food or water had general physiological impacts like reduced total triglycerides, low-density lipoprotein cholesterol, circulating lymphocytes, and viral vaccine-specific antibody titers. Additionally, ceca *Enterobacteriaceae* levels were inconsistently increased or decreased by Gro-2-Max®, regardless the route of inoculation (119). Our team has recently demonstrated changes in chicken intestinal *Enterobacteriaceae* levels via Gro-2-Max®, with layers only fed Gro-2-Max® exhibiting increased *Enterobacteriaceae* fecal shedding compared to the control birds (160). Furthermore, layers showed increased resistance to both APEC and *Salmonella* Kentucky when fed with both live *Salmonella* vaccine and Gro-2-Max® (118), suggesting this probiotic has adjuvant activities.

## Future Directions for Live Prophylactics

Although much progress has been made in protecting poultry against bacterial disease, the movement of poultry animals to cage-free facilities has driven an increase in bacterial infections [reviewed by (3)], which pose risks to both animal and human health. Although the previously-described commercial vaccines and probiotics are used in practice, there are emerging technologies and strategies to improve food safety that warrant discussion. For the duration of this review, we will highlight issues in probiotic label-accuracy, novel yet non-commercial live vaccine strategies, and research gaps where the effects of probiotics and live vaccines are largely-understudied.

### Probiotic Product Label Reliability

Although probiotics are widely-implemented in animal agriculture, label accuracy is a major concern that can drastically-influence product efficacy and health outcomes in poultry animals. More than 28% of the commercial cultures intended for human or animal use were misidentified at the genus or species level through rapid detection methods (161). Looking specifically at poultry probiotics, Redweik and colleagues use PCR to confirm the identification of all probiotic bacteria in Gro-2-Max® but detected *Saccharomyces pastorianus* (not *S. cerevisiae* as advertised) (160). Using four different methods to taxonomically-identify LAB present in FloraMax®-B11, Menconi and colleagues found that each method produced mixed results (93). Although 16S sequencing was the most accurate method used in this study, it is nearly impossible to speciate bacteria via 16S sequencing unless they are highly-characterized [reviewed in (162)], demonstrating its limited application. Using whole-genome shotgun sequencing is a far more accurate tool in addressing current labeling issues and false positive results for species not listed as components (163). Finally, another major concern is the accuracy of bacterial concentrations in these commercial products, as total viable cell counts often do not correspond with the concentrations given on the label (164). Altogether, it is imperative that researchers studying commercial probiotic activities in poultry verify label accuracy to improve repeatability.

### Novel Live Vaccine Strategies

Although live vaccine technologies for *Salmonella*, APEC, *Mycoplasma gallisepticum*, and *Pasteurella multocida* are commercially-available for poultry animals, there is no commercial live vaccines for *Campylobacter* nor *Clostridium* available. *Campylobacter*, a major foodborne pathogen responsible for intestinal and extraintestinal disease in humans (165, 166), typically colonizes the chicken gut as a commensal (167). Despite several studies evaluating the use of whole-cell *Campylobacter* vaccines (168–171) and antigen-based vaccines (172–177), there is no vaccine commercially-available for *Campylobacter* reduction in the intestine. A major issue with orally-delivered, live *Campylobacter* vaccines may arise in distinguishing between vaccine and pathogenic strains during meat processing. To avoid this issue, one solution could be to use another vaccine strain that is genetically-modified to express conserved *Campylobacter* antigens. Several studies have explored the use of *Lactococcus lactis* (178), *Salmonella* (179), and *E. coli* (180) to carry these antigens for anti-*Campylobacter* immune development. However, a major limitation to using antigen-based strategies against *Campylobacter* is that they are highly, antigenically-variable between strains (181), making the identification of a conserved target difficult.

Although necrotic enteritis is a major cause of mortality and reduced productivity in young birds (182, 183), no vaccine is available against its causative agent *Clostridium perfringens*. Non-virulent *C. perfringens* can be used to promote intestinal immunity against pathogenic strains (184). Furthermore, *Salmonella* vaccines carrying recombinant *C. perfringens* antigens have been successful in potent protection against necrotic enteritis (185, 186). Thus, there is much potential for a live, oral vaccine that can protect against *C. perfringens*-induced necrotic enteritis, which might be further-improved through support with probiotics like CylactinΔ, GalliPro®, and CloSTAT® which, on their own, offer protection (96, 101, 102).

### Research Gaps

Most studies evaluate probiotic and live vaccine-efficacy by comparing mono-treated animals vs. non-treated controls. While this experimental design is a crucial first-step in identifying the usefulness of a live prophylactic, this format is not representative of natural commercial conditions and it ignores the impact other vaccines, feed, etc. may have on the animal's response to that live prophylactic of-interest. This is of extreme-importance, as commercial farms routinely use a wide repertoire of prophylactics (live, inactivated, and subunit alike) on their poultry animals without knowing how they might improve or nullify each other's effects. Probiotics are widely-reported to serve as biological, vaccine adjuvants [reviewed in (187)]. However, the role of probiotics in vaccine-responsiveness is largely-understudied in poultry. As mentioned briefly, efficacy and weight gain of a live, recombinant *Campylobacter* vaccine was drastically-improved in broilers which were also given *Anaerosporobacter mobilis* as a probiotic (180). This improvement in vaccine response is even found for live vaccines outside the scope of this review. The protection against the eukaryotic pathogen *Eimeria* was highest when a live coccidiosis vaccine was combined with probiotics (188). Use of Gro-2-MaxΔ in combination with a live *Salmonella* vaccine improved resistance to both intestinal *Salmonella* Kentucky colonization and extraintestinal infection by an O78 APEC (118). This latter study suggests that probiotics can even exert their benefits outside of the intestine, potentially through activation of immune phagocytes via TLR-dependent pathways (126). Another commercial probiotic (Cylactin®) also may be a useful vaccine adjuvant, as combining this product with the live *Salmonella* vaccine Gallivac® SE increased *Salmonella*-specific IgA in layers (97). Thus, it is imperative that future studies look at the synergistic-effect other prophylactics may have on one another. Given the expensive nature of trying to fully-model the spread of prophylactics used in poultry agriculture, one could feasibly use commercially-available birds already given their respective prophylactics prior to experimental treatment.

Although parameters such as weight gain, food-conversion, egg laying efficiency, and bacterial resistance are commonly-used to study prophylactic-efficacy, there are many other mechanisms in which these live microbes could affect the host. Gut bacteria play a major role in the maturation of the enteric nervous system (189) and mediate animal behavior via the gut-brain-microbiota axis (190–192). These interactions are largely-driven by the ability of probiotic bacteria, *Salmonella*, and *E. coli* to directly synthesize and respond-to neurochemicals through a bidirectional communication network called microbial endocrinology (193–195). Animal models have demonstrated the ability for probiotics like *Lactobacillus* and *Bifidobacterium* (196, 197) as well as *C. perfringens* (198) to modulate behavior, although only the latter has been shown in chickens. Recently, a Δ3761-derived *Salmonella* vaccine and Gro-2-MaxΔ were shown to modulate gut catecholamine (but not serotonin) metabolism in layer pullets, depending if the live prophylactics were given individually or in combination (160). Altogether, these findings suggest that the prophylactics used may have a direct impact on animal behavior. Thus, a novel target for live prophylactics could be to manipulate poultry animals into exhibiting positive behaviors (feeding, dust-bathing) while mitigating negative social behaviors like pecking. However, a major consideration is whether effects of these live prophylactics on the gut-brain-microbiota axis are maintained by chickens with different gut microbiotas. Given the variability of the chicken gut microbiome due to factors like geographical location, litter, breed, and feed [reviewed by (199)], it is very possible that other commensal bacteria might nullify, reduce, or amplify the effects live prophylactics might have on animal behavior and neurochemical metabolism.

## Conclusions

**Figure 1:**
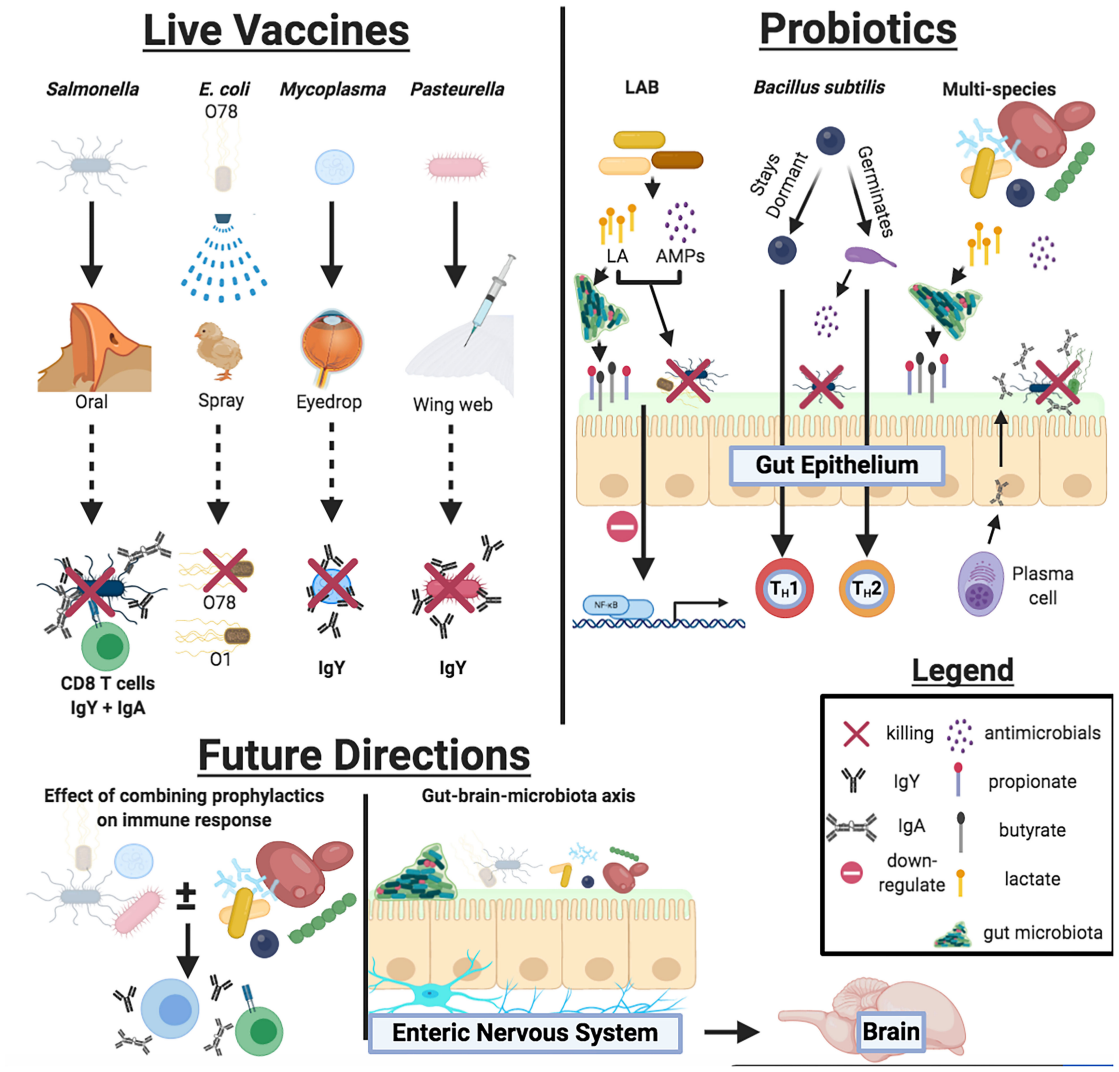
Overview of mechanisms live bacterial vaccines and probiotics participate in to improve host health, responses against bacterial pathogens, and future directions for live prophylactic research.

Commercial live bacterial vaccines and probiotics offer several advantages in improving poultry health against bacterial disease and colonization (summarized in Figure 1) However, a paucity of peer-reviewed research studies, inconsistencies with product labels, limited cross-protection against certain pathogens, and a vague understanding of synergistic effects when using multiple prophylactics have encumbered our ability to optimize poultry health. Additionally, it is crucial that future studies must investigate whether these live prophylactics may facilitate animal behavior changes via the gut-brain axis (Figure 1), providing a convenient means of improving social behaviors among poultry flock.

## Author Contributions

GR and JJ wrote manuscript and developed figures and tables. MM revised manuscript. GR and MM provided funding and conceptualized review topic. All authors contributed to the article and approved the submitted version.

## Conflict of Interest

The authors declare that the research was conducted in the absence of any commercial or financial relationships that could be construed as a potential conflict of interest.

## References

[1] Food and Agriculture Organization of the United Nations. FAOSTAT: Live Animals Data. (2017). Available online from: http://www.fao.org/faostat/en

[2] USDA Agricultural Marketing Service. (2019) (accessed June 9, 2020).

[3] Lay DC Fulton RM Hester PY Karcher DM Kjaer JB Mench JA. Hen welfare in different housing systems. Poult Sci. (2011) 90:278–94. 10.3382/ps.2010-00962 21177469

[4] Uddin J Hossain K Hossain S Saha K Jubyda FT Haque R. Bacteriological assessments of foodborne pathogens in poultry meat at different super shops in Dhaka, Bangladesh. Ital J Food Saf. (2019) 8:6720. 10.4081/ijfs.2019.6720 31008079 PMC6452097

[5] Heredia N García S. Animals as sources of food-borne pathogens: a review. Anim Nutr. (2018) 4:250–5. 10.1016/j.aninu.2018.04.006 30175252 PMC6116329

[6] Wegener HC Antibiotic resistance- linking human and animal health. In: *Institute of Medicine (US). Improving Food Safety Through a One Health Approach: Workshop Summary*. Washington, DC: National Academies Press (2012). p. A15. Available online at: https://www.ncbi.nlm.nih.gov/books/NBK114485/23230579

[7] Tadesse DA Zhao S Tong E Ayers S Singh A Bartholomew MJ. Antimicrobial drug resistance in *Escherichia coli* from humans and food animals, United States, 1950-2002. Emerg Infect Dis. (2012) 18:741–9. 10.3201/eid1805.111153 22515968 PMC3358085

[8] Simoneit C Burow E Tenhagen BA Käsbohrer A. Oral administration of antimicrobials increase antimicrobial resistance in *E. coli* from chicken- a systematic review. Prev Vet Med. (2015) 118:1–7. 10.1016/j.prevetmed.2014.11.010 25433717

[9] Kheiri R Akhtari L. Antimicrobial resistance and integron gene cassette arrays in commensal *Escherichia coli* from human and animal sources in IRI. Gut Pathog. (2016) 8:40. 10.1186/s13099-016-0123-3 27582900 PMC5006490

[10] Nhung NT Chansiripornchai N Carrique-Mas JJ. Antimicrobial resistance in bacterial poultry pathogens: a review. Front Vet Sci. (2017) 4:126. 10.3389/fvets.2017.00126 28848739 PMC5554362

[11] Wigley P. *Salmonella enterica* in the chicken: how it has helped our understanding of immunology in a non-biomedical model species. Front Immunol. (2014) 5:482. 10.3389/fimmu.2014.00482 25346731 PMC4193332

[12] Wilson DJ Gabriel E Leatherbarrow AJ Cheesbrough J Gee S Bolton E. Tracing the source of campylobacteriosis. PLoS Genet. (2008) 4:e1000203. 10.1371/journal.pgen.1000203 18818764 PMC2538567

[13] Antunes P Mourão J Campos J Peixe L. Salmonellosis: the role of poultry meat. Clin Microbiol Infect. (2016) 22:110–21. 10.1016/j.cmi.2015.12.004 26708671

[14] Friedman CR Neimann J Wegener HC Tauxe RV Epidemiology of *Campylobacter jejuni* infections in the United States and other industrialized nations. In: Nachamkin I, Blaser MJ, editors. Campylobacter. 2nd ed. Washington, DC: American Society for Microbiology (2000). p. 121–138.

[15] European food safety authority and european centre for disease prevention and control The European union summary report on trends and sources of zoonoses, zoonotic agents and food-borne outbreaks in 2014. EFSA J. (2015) 13:4329–8. 10.2903/j.efsa.2015.4329

[16] Fricke WF McDermott PF Mammel MK Zhao S Johnson TJ Rasko DA. Antimicrobial resistance-conferring plasmids with similarity to virulence plasmids from avian pathogenic *Escherichia coli* strains in *Salmonella enterica* serovar kentucky isolates from poultry. Appl Environ Microbiol. (2009) 75:5963–71. 10.1128/AEM.00786-09 19648374 PMC2747853

[17] Card RM Cawthraw SA Nunez-Garcia J Ellis RJ Kay G Pallen MJ. An *in vitro* chicken gut model demonstrates transfer of a multidrug resistance plasmid from *Salmonella* to commensal *Escherichia coli*. mBio. (2017) 8:00777–17. 10.1128/mBio.00777-17 28720731 PMC5516254

[18] Avrain L Vernozy-Rozand C Kempf I. Evidence for natural horizontal transfer of *tetO* gene between *Campylobacter jejuni* strains in chickens. J Appl Microbiol. (2004) 97:134–40. 10.1111/j.1365-2672.2004.02306.x 15186450

[19] Rabie NS Amin Girh ZMS. Bacterial vaccines in poultry. Bull Natl Res Cent. (2020) 44:15. 10.1186/s42269-019-0260-1 32435127 PMC7223244

[20] Hill C Guarner F Reid G Gibson GR Merenstein DJ Pot B. Expert consensus document. The international scientific association for probiotics and prebiotics consensus statement on the scope and appropriate use of the term probiotic. Nat Rev Gastroenterol Hepatol. (2014) 11:506–14. 10.1038/nrgastro.2014.66 24912386

[21] Lin IY Van TT Smooker PM. Live-attenuated bacterial vectors: tools for vaccine and therapeutic agent delivery. Vaccines. (2015) 3:940–72. 10.3390/vaccines3040940 26569321 PMC4693226

[22] Fenster K Freeburg B Hollard C Wong C Rønhave Laursen R Ouwehand AC. The production and delivery of probiotics: a review of a practical approach. Microorganisms. (2019) 7:83. 10.3390/microorganisms7030083 30884906 PMC6463069

[23] Detmer A Glenting J. Live bacterial vaccines- a review and identification of potential hazards. Microb Cell Fact. (2006) 5:23. 10.1186/1475-2859-5-23 16796731 PMC1538998

[24] Clark-Curtiss JE Curtiss R. Vaccines: conduits for protective antigens. J Immunol. (2018) 200:39–48. 10.4049/jimmunol.1600608 29255088

[25] Kennedy DA Read AF. Why does drug resistance readily evolve but vaccine resistance does not? Proc Biol Sci. (2017) 284:20162562. 10.1098/rspb.2016.2562 28356449 PMC5378080

[26] Hassan JO Curtiss R. Virulent *Salmonella typhimurium*-induced lymphocyte depletion and immunosuppression in chickens. Infect Immun. (1994) 62:2027–36. 10.1128/IAI.62.5.2027-2036.1994 8168969 PMC186462

[27] McReynolds JL Moore RW McElroy AP Hargis BM Caldwell DJ Evaluation of a competitive exclusion culture and megan Vac 1 on *Salmonella Typhimurium* colonization in neonatal broiler chickens. J Appl Poult Res. (2007) 16:456–63. 10.1093/japr/16.3.456

[28] Dórea FC Cole DJ Hofacre C Zamperini K Mathis D Doyle MP. Effect of *Salmonella* vaccination of breeder chickens on contamination of broiler chicken carcasses in integrated poultry operations. Appl Environ Microbiol. (2010) 76:7820–5. 10.1128/AEM.01320-10 20889797 PMC2988591

[29] Bailey JS Rolon A Hofacre CL Holt PS Wilson JL Cosby DE Intestinal humoral immune response and resistance to *Salmonella* challenge of progeny from breeders vaccinated with killed antigen. Int J Poult Sci. (2007) 6:417–23. 10.3923/ijps.2007.417.423

[30] Hayashi RM Tujimoto-Silva A Muniz EC Verdi R Santin E *Salmonella Typhimurium* vaccine to control a brazilian *Salmonella* heidelberg strain in broiler chickens. ARS Vet. (2018) 34:105–14. 10.15361/2175-0106.2018v34n3p105-114

[31] Muniz EC Verdi R Leão JA Back A Nascimento VPD. Evaluation of the effectiveness and safety of a genetically modified live vaccine in broilers challenged with *Salmonella* Heidelberg. Avian Pathol. (2017) 46:676–82. 10.1080/03079457.2017.1348598 28660788

[32] Springer S Lindner T Ahrens M Woitow G Prandini F Selbitz HJ. Duration of immunity induced in chickens by an attenuated live *Salmonella* enteritidis vaccine and an inactivated *Salmonella enteritidis/typhimurium* vaccine. Berl Munch Tierarztl Wochenschr. (2011) 124:89–93. 21462861

[33] Campagnari E Rossi G Franciosi C Girelli D Giovanardi D *In vitro* evaluation of live attenuated vaccines against *Salmonella enteritidis*: cell-mediated immune response. Italian J Anim Sci. (2007) 6:301–4. 10.4081/ijas.2007.301

[34] Sadeyen J Wu Z Davies H. Immune responses associated with homologous protection conferred by commercial vaccines for control of avian pathogenic *Escherichia coli* in turkeys. Vet Res. (2015) 46:5. 10.1186/s13567-014-0132-5 25613193 PMC4304773

[35] Mohamed MA Bakhit BM Ibrahim AA Saleh M Evaluation of the living *Escherichia coli*-O78 deleted aroA vaccine against homologous and heterologous *E. coli* challenge in broiler chickens. J Adv Vet Res. (2016) 6:89–92.

[36] Bíró J Povazsán J Korösi L Glávits R Hufnagel L Stipkovits L. Safety and efficacy of *Mycoplasma gallisepticum* TS-11 vaccine for the protection of layer pullets against challenge with virulent *M. gallisepticum* R-strain. Avian Pathol. (2005) 34:341–7. 10.1080/03079450500179913 16147571

[37] Branton SL Lott BD May JD Maslin WR Pharr GT Bearson SD. The effects of ts-11 *strain Mycoplasma gallisepticum* vaccination in commercial layers on egg production and selected egg quality parameters. Avian Dis. (2000) 44:618–23. 11007009

[38] Leigh SA Evans JD Branton SL Collier SD. The effects of increasing sodium chloride concentration on *Mycoplasma gallisepticum* vaccine survival in solution. Avian Dis. (2008) 52:136–8. 10.1637/7979-040507-ResNote 18459310

[39] Evans JD Leigh SA Branton SL Collier SD. Effects of increased dosages of the *Mycoplasma gallisepticum* vaccine MYCOVAC-L in layer chickens subsequently challenged with virulent *M. gallisepticum*: egg production and serologic response. Avian Dis. (2007) 51:912–7. 10.1637/7931-020807-REGR2.1 18251402

[40] Evans JD Leigh SA Purswell JL Jacob R Peebles ED Collier SD. A comparative study of live attenuated F strain-derived *Mycoplasma gallisepticum* vaccines. Avian Dis. (2012) 56:396–401. 10.1637/9951-092711-Reg.1 22856200

[41] Leigh SA Evans ED Collier SD Branton SL. The impact of vaccination route on *Mycoplasma gallisepticum* vaccine efficacy. Poult Sci. (2018) 97:3072–5. 10.3382/ps/pey188 29788205

[42] Evans JD Jacob R Leigh SA Collier SD Peebles ED Branton SL. Spray application of live attenuated F strain-derived *Mycoplasma gallisepticum* vaccines. J Appl Poult Res. (2013) 22:842–8. 10.3382/japr.2013-0074922856200

[43] Peebles ED Jacob R Branton SL Evans JD Leigh SA Gerard PD Effects of different vaccine combinations against *Mycoplasma gallisepticum* on blood characteristics in commercial layer chickens. Poult Sci. (2015) 94:2108–13. 10.3382/ps/pev225 26217033

[44] Umar S Munir MT Rehman Z Subhan S Azam T Shah MAA Mycoplasmosis in poultry: update on diagnosis and preventive measures. Poult Sci J. (2017) 73:1–12. 10.1017/S0043933916000830

[45] Sharaf MM Nestor KE Saif YM Sacco RE Havenstein GB. Antibody response to Newcastle disease virus and *Pasteurella multocida* of two strains of turkeys. Poult Sci. (1988) 67:1372–7. 10.3382/ps.0671372 3194331

[46] Berndt A Wilhelm A Jugert C Pieper J Sachse K. Chicken cecum immune response to *Salmonella enterica* serovars of different levels of invasiveness. Infect Immun. (2007) 75:5993–6007. 10.1128/IAI.00695-07 17709416 PMC2168364

[47] Setta AM Barrow PA Kaiser P Jones MA. Early immune dynamics following infection with Salmonella enterica serovars enteritidis, infantis, pullorum and gallinarum: cytokine and chemokine gene expression profile and cellular changes of chicken cecal tonsils. Comp Immunol Microbiol Infect Dis. (2012) 35:397–410. 10.1016/j.cimid.2012.03.004 22512820

[48] Varmuzova K Matulova ME Sebkova A Sekelova Z Havlickova H. The early innate response of chickens to Salmonella enterica is dependent on the presence of o-antigen but not on serovar classification. PLoS ONE. (2014) 9:e96116. 10.1371/journal.pone.0096116 24763249 PMC3999269

[49] Batz MB Hoffmann S Morris JG. Ranking the disease burden of 14 pathogens in food sources in the United States using attribution data from outbreak investigations and expert elicitation. J Food Prot. (2012) 75:1278–91. 10.4315/0362-028X.JFP-11-418 22980012

[50] Center for Disease Control and Prevention (2020). Salmonella Homepage. Avaialble online at: https://www.cdc.gov/salmonella/inde.html (accessed June 16, 2020).

[51] Pei Y Parreira V Roland K Curtiss R Prescott J. Assessment of attenuated *Salmonella* vaccine strains in controlling experimental *Salmonella* Typhimurium infection in chickens. Can J Vet Res. (2014) 78:23–30. 24396177 PMC3878005

[52] Pei Y Parreira V Roland K Curtiss R Prescott J. Assessment of attenuated *Salmonella* vaccine strains in controlling experimental *Salmonella* Typhimurium infection in chickens. Can J Vet Res. (2014) 78:23–30. 24396177 PMC3878005

[53] Juárez-Rodríguez MD Arteaga-Cortés LT Kader R Curtiss R 3rd Clark-Curtiss JE Live attenuated *Salmonella* vaccines against *Mycobacterium tuberculosis* with antigen delivery via the type III secretion system. Infect Immun. (2012) 80:798–814. 10.1128/IAI.05525-11 22144486 PMC3264309

[54] Chaudhari AA Matsuda K Lee JH. Construction of an attenuated *Salmonella* delivery system harboring genes encoding various virulence factors of avian pathogenic *Escherichia coli* and its potential as a candidate vaccine for chicken colibacillosis. Avian Dis. (2013) 57:88–96. 10.1637/10277-061312-Reg.1 23678735

[55] Luo Y Kong Q Yang J Mitra A Golden G Wanda SY. Comparative genome analysis of the high pathogenicity *Salmonella Typhimurium* strain UK-1. PLoS ONE. (2012) 7:e40645. 10.1371/journal.pone.0040645 22792393 PMC3391293

[56] Withanage GS Wigley P Kaiser P Mastroeni P Brooks H Powers C. Cytokine and chemokine responses associated with clearance of a primary *Salmonella enterica* serovar *Typhimurium* infection in the chicken and in protective immunity to rechallenge. Infect Immun. (2005) 73:5173–82. 10.1128/IAI.73.8.5173-5182.2005 16041035 PMC1201213

[57] Mellata M. Human and avian extraintestinal pathogenic *Escherichia coli*: infections, zoonotic risks, and antibiotic resistance trends. Foodborne Pathog Dis. (2013) 10:916–32. 10.1089/fpd.2013.1533 23962019 PMC3865812

[58] Rolain JM. Food and human gut as reservoirs of transferable antibiotic resistance encoding genes. Front Microbiol. (2013) 4:173. 10.3389/fmicb.2013.00173 23805136 PMC3690338

[59] Johnson TJ Siek KE Johnson SJ Nolan LK. DNA sequence and comparative genomics of pAPEC-O2-R, an avian pathogenic *Escherichia coli* transmissible R plasmid. Antimicrob Agents Chemother. (2005) 49:4681–8. 10.1128/AAC.49.11.4681-4688.2005 16251312 PMC1280136

[60] Yang QE Sun J Li L Deng H Liu BT Fang LX. IncF plasmid diversity in multi-drug resistant *Escherichia coli* strains from animals in China. Front Microbiol. (2015) 6:964. 10.3389/fmicb.2015.00964 26441898 PMC4585273

[61] Johnson TJ Thorsness JL Anderson CP Lynne AM Foley SL Han J. Horizontal gene transfer of a ColV plasmid has resulted in a dominant avian clonal type of *Salmonella enterica* serovar kentucky. PLoS ONE. (2010) 5:e15524. 10.1371/journal.pone.0015524 21203520 PMC3008734

[62] Solà-Ginés M Cameron-Veas K Badiola I Dolz R Majó N Dahbi G. Diversity of multi-drug resistant avian pathogenic *Escherichia coli* (APEC) causing outbreaks of colibacillosis in broilers during 2012 in Spain. PLoS ONE. (2015) 10:e0143191. 10.1371/journal.pone.0143191 26600205 PMC4657910

[63] Ewers C Antão EM Diehl I Philipp HC Wieler LH. Intestine and environment of the chicken as reservoirs for extraintestinal pathogenic *Escherichia coli* strains with zoonotic potential. Appl Environ Microbiol. (2009) 75:184–92. 10.1128/AEM.01324-08 18997030 PMC2612213

[64] Stromberg ZR Johnson JR Fairbrother JM Kilbourne J Van Goor A Curtiss R. Evaluation of *Escherichia coli* isolates from healthy chickens to determine their potential risk to poultry and human health. PLoS ONE. (2017) 12:e0180599. 10.1371/journal.pone.0180599 28671990 PMC5495491

[65] Duan H Chai T Cai Y Zhong Z Yao M Zhang X. Transmission identification of *Escherichia coli* aerosol in chicken houses to their environments using ERIC-PCR. Sci China C Life Sci. (2008) 51:164–73. 10.1007/s11427-008-0021-0 18239895 PMC7089447

[66] Chien YC Chen CJ Lin TH Chen SH. Characteristics of microbial aerosols released from chicken and swine feces. J Air Waste Manag Assoc. (2011) 61:882–9. 10.3155/1047-3289.61.8.882 21874960

[67] Sadeghi AA Shawrang P Shakorzadeh S. Immune response of *Salmonella* challenged broiler chickens fed diets containing gallipro®, a *Bacillus subtilis* probiotic. Probiotics Antimicrob Proteins. (2015) 7:24–30. 10.1007/s12602-014-9175-1 25344127

[68] Evans JD Leigh SA Branton SL Collier SD Pharr GT Bearson SMD *Mycoplasma gallisepticum*: current and developing means to control the avian pathogen. J Appl Poult Res. (2005) 14:757–63. 10.1093/japr/14.4.757

[69] Shoaib M Mycoplasmosis in poultry, a perpetual problem. J Microbiol Biotechnol Food Sci. (2019) 8:1271–5. 10.15414/jmbfs.2019.8.6.1271-1275

[70] Stipkovits L Kempf I. Mycoplasmoses in poultry. Rev Sci Tech. (1996) 15:1495–525. 10.20506/rst.15.4.986 9190023

[71] Charleston B Gate JJ Aitken IA Reeve-Johnson L. Assessment of the efficacy of tilmicosin as a treatment for *Mycoplasma gallisepticum* infections in chickens. Avian Pathol. (1998) 27:190–5. 10.1080/03079459808419322 18483985

[72] Patterson PH Coping with *Mycoplasma gallisepticum*. Internews. (1994) 7:1–3.

[73] Mohammed HO Carpenter TE Yamamoto R. Economic impact of My*coplasma gallisepticum* and *M*. synoviae in commercial layer flocks. Avian Dis. (1987) 31:477–82. 10.2307/1590727 3675423

[74] Levisohn S Dykstra MJ. A quantitative study of single and mixed infection of the chicken trachea by *Mycoplasma gallisepticum*. Avian Dis. (1987) 31:1–12. 10.2307/1590765 3579778

[75] Minion FC. Molecular pathogenesis of mycoplasma animal respiratory pathogens. Front Biosci. (2002) 7:d1410–22. 10.2741/A849 12045010

[76] Kulappu Arachchige SN Young ND Shil PK Legione AR Kanci Condello A Browning GF. Differential response of the chicken trachea to chronic infection with virulent *Mycoplasma gallisepticum* strain Ap3AS and vaxsafe MG (Strain ts-304): a transcriptional profile. Infect Immun. (2020) 88:e00053–20. 10.1128/IAI.00053-20 32122943 PMC7171234

[77] Zhang W Liu Y Zhang Q Waqas Ali Shah S Wu Z Wang J. Infection impaired the structural integrity and immune function of bursa of fabricius in chicken: implication of oxidative stress and apoptosis. Front Vet Sci. (2020) 7:225. 10.3389/fvets.2020.00225 32391391 PMC7193947

[78] Nunoya T Yagihashi T Tajima M Nagasawa Y. Occurrence of keratoconjunctivitis apparently caused by *Mycoplasma gallisepticum* in layer chickens. Vet Pathol. (1995) 32:11–8. 10.1177/030098589503200102 7725593

[79] Leigh SA Evans JD Branton SL. Complete genome sequences of two vaccine strains and one field isolate of *Mycoplasma gallisepticum*. Microbiol Resour Announc. (2019) 8:e01237–19. 10.1128/MRA.01237-19 31806746 PMC6895306

[80] Shil PK Kanci A Browning GF Marenda MS Noormohammadi AH Markham PF. GapA+ *Mycoplasma gallisepticum* ts-11 has improved vaccine characteristics. Microbiology. (2011) 157:1740–9. 10.1099/mic.0.046789-0 21310786

[81] Leigh SA Branton SL Collier SD Evans JD Analysis of the effect of diluent for rehydration of poulvac® MycoF on vaccination seroconversion results. Int J Poult Sci. (2011) 10:397–400. 10.3923/ijps.2011.397.400

[82] Purswell JL Evans JD Leigh SA Collier SD Olanrewaju HA. *Mycoplasma gallisepticum* transmission: comparison of commercial F-strain vaccine versus layer complex-derived field strains in a tunnel ventilated house. Poult Sci. (2012) 91:3072–9. 10.3382/ps.2012-02619 23155015

[83] Glisson JR Hofacre CL Christensen JP Fowl cholera. In: Saif YM, Fadly AM, editors. Diseases of Poultry. 12th ed. Ames, IA: Blackwell Publishing (2008). p. 739–58.

[84] Boyce JD Harper M Wilkie IW Adler B Pasteurella. In: Gyles CL, Prescott JF, Songer JG, Thoen CO, editors. Pathogenesis of Bacterial Infections in Animals. 4th ed. Ames, IA: Wiley-Blackwell (2010). p. 325–46.

[85] Petersen KD Christensen JP Permin A Bisgaard M. Virulence of *Pasteurella multocida* subsp. multocida isolated from outbreaks of fowl cholera in wild birds for domestic poultry and game birds. Avian Pathol. (2001) 30:27–31. 10.1080/03079450020023168 19184870

[86] Singh R Remington B Blackall P Turni C. Epidemiology of fowl cholera in free range broilers. Avian Dis. (2014) 58:124–8. 10.1637/10656-090313-Reg.1 24758124

[87] OBS Commission Editor Fowl Cholera, in Manual of Diagnostic Tests and Vaccines for Terrestrial Animals. Paris: World Organisation for Animal Health (OIE) (2012). p. 500–506.

[88] Harper M John M Edmunds M Wright A Ford M Turni C. Protective efficacy afforded by live *Pasteurella multocida* vaccines in chickens is independent of lipopolysaccharide outer core structure. Vaccine. (2016) 34:1696–703. 10.1016/j.vaccine.2016.02.017 26892738

[89] Taylor KJM Ngunjiri JM Abundo MC Jang H Elaish M Ghorbani A. Respiratory and gut microbiota in commercial turkey flocks with disparate weight gain trajectories display differential compositional dynamics. Appl Environ Microbiol. (2020) 86:e00431–20. 10.1128/AEM.00431-20 32276973 PMC7267191

[90] Sander JE Resurreccion RS Waltman WD McMurray BL. *Pasteurella* challenge and ELISA serology evaluation of broiler breeders vaccinated with live cholera vaccine. Avian Dis. (1998) 42:190–3. 10.2307/1592595 9533100

[91] Higgins SE Wolfenden AD Tellez G Hargis BM Porter TE. Transcriptional profiling of cecal gene expression in probiotic- and *Salmonella*-challenged neonatal chicks. Poult Sci. (2011) 90:901–13. 10.3382/ps.2010-00907 21406379

[92] Prado-Rebolledo OF Delgado-Machuca JDJ Macedo-Barragan RJ Garcia-Márquez LJ Morales-Barrera JE Latorre JD Evaluation of a selected lactic acid bacteria-based probiotic on *Salmonella enterica* serovar enteritidis colonization and intestinal permeability in broiler chickens. Avian Pathol. (2017) 46:90–4. 10.1080/03079457.2016.1222808 27545145

[93] Menconi A Kallapura G Latorre JD Morgan MJ Pumford NR Hargis BM. Identification and characterization of lactic acid bacteria in a commercial probiotic culture. Biosci Microbiota Food Health. (2014) 33:25–30. 10.12938/bmfh.33.25 24936379 PMC4034328

[94] Biloni A Quintana CF Menconi A Kallapura G Latorre J Pixley C. Evaluation of effects of earlybird associated with floramax-B11 on *Salmonella* enteritidis, intestinal morphology, and performance of broiler chickens. Poult Sci. (2013) 92:2337–46. 10.3382/ps.2013-03279 23960116

[95] Layton SL Hernandez-Velasco X Chaitanya S Xavier J Menconi A Latorre JD The effect of a *Lactobacillus*-based probiotic for the control of necrotic enteritis in broilers. Food Nutr Sci. (2013) 4:1–7. 10.4236/fns.2013.411A001

[96] Slizewska K Markowiak-Kopeć P Zbikowski A Szeleszczuk P. The effect of synbiotic preparations on the intestinal microbiota and her metabolism in broiler chickens. Sci Rep. (2020) 10:4281. 10.1038/s41598-020-61256-z 32152423 PMC7062770

[97] Beirão BCB Ingberman M Favaro C Jr Mesa D Bittencourt LC Fascina VB. Effect of an *Enterococcus faecium* probiotic on specific IgA following live *Salmonella* enteritidis vaccination of layer chickens. Avian Pathol. (2018) 47:325–33. 10.1080/03079457.2018.1450487 29534604

[98] Upadhaya SD Rudeaux F Kim IH. Effects of inclusion of *Bacillus subtilis* (Gallipro) to energy- and protein-reduced diet on growth performance, nutrient digestibility, and meat quality and gas emission in broilers. Poult Sci. (2019) 98:2169–78. 10.3382/ps/pey573 30615142

[99] Knap I Kehlet AB Bennedsen M Mathis GF Hofacre CL Lumpkins BS. *Bacillus subtilis* (DSM17299) significantly reduces *Salmonella* in broilers. Poult Sci. (2011) 90:1690–4. 10.3382/ps.2010-01056 21753205

[100] Zaghari M Zahroojian N Riahi M Parhizkar S Effect of *Bacillus Subtilis* spore (GalliPro®) nutrients equivalency value on broiler chicken performance. Italian J Anim Sci. (2015) 14:3555 10.4081/ijas.2015.3555

[101] Abudabos AM Al-Batshan HA Murshed MA Effects of prebiotics and probiotics on the performance and bacterial colonization of broiler chickens. South Afr J Anim Sci. (2015) 45:419 10.4314/sajas.v45i4.8

[102] Abudabos AM Alyemni AH *Bacillus subtilis* PB6 based-probiotic (CloSTATTM) improves intestinal morphological and microbiological status of broiler chickens under *Clostridium perfringens* challenge. Int J Agric Biol. (2013) 15:973–82.

[103] Teo AYL Tan HM Effect of *Bacillus subtilis* PB6 (CloSTAT) on broilers infected with a pathogenic strain of *Escherichia coli*. J Appl Poult Res. (2006) 15:229–35. 10.1093/japr/15.2.229

[104] Latorre JD Hernandez-Velasco X Wolfenden RE Vicente JL Wolfenden AD Menconi A. Evaluation and selection of bacillus species based on enzyme production, antimicrobial activity, and biofilm synthesis as direct-fed microbial candidates for poultry. Front Vet Sci. (2016) 3:95. 10.3389/fvets.2016.00095 27812526 PMC5071321

[105] Hernandez-Patlan D Solis-Cruz B Pontin KP Hernandez-Velasco X Merino-Guzman R Adhikari B. Impact of a *Bacillus* direct-fed microbial on growth performance, intestinal barrier integrity, necrotic enteritis lesions, and ileal microbiota in broiler chickens using a laboratory challenge model. Front Vet Sci. (2019) 6:108. 10.3389/fvets.2019.00108 31106209 PMC6492466

[106] Adhikari B Hernandez-Patlan D Solis-Cruz B Kwon YM Arreguin MA Latorre JD. Evaluation of the antimicrobial and anti-inflammatory properties of bacillus-DFM (NorumTM) in broiler chickens infected with Salmonella enteritidis. Front Vet Sci. (2019) 6:282. 10.3389/fvets.2019.00282 31508436 PMC6718558

[107] Tellez G Arreguin-Nava MA Maguey JA Michel MA Latorre JD Merino-Guzman R. Effect of Bacillus-direct-fed microbial on leaky gut, serum peptide YY concentration, bone mineralization, and ammonia excretion in neonatal female turkey poults fed with a rye-based diet. Poult Sci. (2020) 99:4514–20. 10.1016/j.psj.2020.06.018 32867995 PMC7598103

[108] Arreguin-Nava MA Graham BD Adhikari B Agnello M Selby CM Hernandez-Velasco X. Evaluation of *in ovo Bacillus* spp. based probiotic administration on horizontal transmission of virulent *Escherichia coli* in neonatal broiler chickens. Poult Sci. (2019) 98:6483–91. 10.3382/ps/pez544 31549175 PMC8913981

[109] Smialek M Burchardt S Koncicki A. The influence of probiotic supplementation in broiler chickens on population and carcass contamination with *Campylobacter* spp. Res Vet Sci. (2018) 118:312–6. 10.1016/j.rvsc.2018.03.009 29567598

[110] Bogucka J Ribeiro DM Bogusławska-Tryk M Dankowiakowska A Costa RPR da Bednarczyk M Microstructure of the small intestine in broiler chickens fed a diet with probiotic or synbiotic supplementation. J Anim Physiol Anim Nutr. (2019) 103:1785–91. 10.1111/jpn.13182 31553085

[111] Grimes JL Rahimi S Oviedo E Sheldon BW Santos FBO. Effects of a direct-fed microbial (Primalac) on turkey poult performance and susceptibility to oral *Salmonella* challenge. Poult Sci. (2008) 87:1464–70. 10.3382/ps.2008-00498 18577631

[112] Naseri K Rahimi S Khaki P Comparison of the effects of probiotic, organic acid, and medicinal plant on *Campylobacter jejuni* challenged broiler chickens. J Agricult Sci Tech. (2012) 14:1485–96.

[113] Embrahimi H Rahimi S Khaki P The effect of organic acid, probiotic and *Echinacea purpurea* usage on gastrointestinal microflora and immune system in broiler chickens. J Vet Res. (2015) 70:293–9.

[114] Abudabos AM Effect of primalac® or enramycin supplementation on performance, intestinal morphology and microbiology of broilers under *Clostridium perfringens* challenge. J Food Agric Environ. (2012) 10:595-9.

[115] Embrahimi H Rahimi S Khaki P Grimes JL Kathariou S The effects of probiotics, organic acid, and a medicinal plant on the immune system and gastrointestinal microflora in broilers challenged with *Campylobacter jejuni*. Turk J Vet Anim Sci. (2016) 40:329–36. 10.3906/vet-1502-68

[116] Pender CM Kim S Potter TD Ritzi MM Young M Dalloul RA. In ovo supplementation of probiotics and its effects on performance and immune-related gene expression in broiler chicks. Poult Sci. (2017) 96:1052–62. 10.3382/ps/pew381 28158826

[117] Manafi M Hedayati M Mirzaie S Probiotic *Bacillus* species and *Saccharomyces boulardii* improve performance, gut histology and immunity in broiler chickens. South Afr J Anim Sci. (2018) 48:379–89. 10.4314/sajas.v48i2.19

[118] Redweik GAJ Daniels K Severin AJ Lyte M Mellata M. Oral treatments with probiotics and live *Salmonella* vaccine induce unique changes in gut neurochemicals and microbiome in chickens. Front Microbiol. (2020) 10:3064. 10.3389/fmicb.2019.03064 32010110 PMC6974472

[119] Abd El-Baky AA Shalaby NA Abdelgayed SS Assessment of growth performance, hemato-biochemical parameters, immunological and histopathological alterations associated with new bacterial multistrain probiotic (Gro-2-Max) supplementation on broiler chicken. Int J ChemTech Res. (2016) 9:996–1016.

[120] Liao SF Nyachoti M. Using probiotics to improve swine gut health and nutrient utilization. Anim Nutr. (2017) 3:331–43. 10.1016/j.aninu.2017.06.007 29767089 PMC5941265

[121] Russo P Arena MP Fiocco D Capozzi V Drider D Spano G. *Lactobacillus plantarum* with broad antifungal activity: a promising approach to increase safety and shelf-life of cereal-based products. Int J Food Microb. (2017) 247:48–54. 10.1016/j.ijfoodmicro.2016.04.027 27240933

[122] Dittoe DK Ricke SC Kiess AS. Organic acids and potential for modifying the avian gastrointestinal tract and reducing pathogens and disease. Front Vet Sci. (2018) 5:216. 10.3389/fvets.2018.00216 30238011 PMC6136276

[123] Zhitnitsky D Rose J Lewinson O. The highly synergistic, broad spectrum, antibacterial activity of organic acids and transition metals. Sci Rep. (2017) 7:44554. 10.1038/srep44554 28294164 PMC5353632

[124] Vieco-Saiz N Belguesmia Y Raspoet R Auclair E Gancel F Isabelle K. Benefits and inputs from lactic acid bacteria and their bacteriocins as alternatives to antibiotic growth promoters during food-animal production. Front Microbiol. (2019) 10:57. 10.3389/fmicb.2019.00057 30804896 PMC6378274

[125] Ren C Cheng L Sun Y Zhang Q De Haan BJ Zhang H Lactic acid bacteria secrete toll like receptor 2 stimulating and macrophage immunomodulating bioactive factors. J Funct Foods. (2020) 66:103783 10.1016/j.jff.2020.103783

[126] DvoroŽnáková E Bucková B Hurníková Z Revajová V Lauková A. Effect of probiotic bacteria on phagocytosis and respiratory burst activity of blood polymorphonuclear leukocytes (PMNL) in mice infected with *Trichinella spiralis*. Vet Parasitol. (2016) 231:69–76. 10.1016/j.vetpar.2016.07.004 27425573

[127] Kogut MH Tellez GI McGruder ED Hargis BM Williams JD Corrier DE. Heterophils are decisive components in the early responses of chickens to *Salmonella* enteritidis infections. Microb Pathog. (1994) 16:141–51. 10.1006/mpat.1994.1015 8047002

[128] Mirza RA Probiotics and prebiotics for the health of poultry. in Probiotics and Prebiotics in Animal Health and Food Safety. Erbil: Springer International Publishing (2018). p. 127–54.

[129] Bednorz C Guenther S Oelgeschläger K Kinnemann B Pieper R Hartmann S. Feeding the probiotic *Enterococcus faecium* strain NCIMB 10415 to piglets specifically reduces the number of *Escherichia coli* pathotypes that adhere to the gut mucosa. Appl Environ Microbiol. (2013) 79:7896–904. 10.1128/AEM.03138-13 24123741 PMC3837809

[130] Penaloza-Vazquez A Ma LM Rayas-Duarte P. Isolation and characterization of *Bacillus* spp. Can J Microbiol. (2019) 65:762–74. 10.1139/cjm-2019-0019 31393167

[131] La Ragione RM Woodward MJ. Competitive exclusion by *Bacillus subtilis* spores of *Salmonella enterica* serotype enteritidis and *Clostridium perfringens* in young chickens. Vet Microbiol. (2003) 94:245–56. 10.1016/S0378-1135(03)00077-4 12814892

[132] Whelan RA Doranalli K Rinttilä T Vienola K Jurgens G Apajalahti J. The impact of *Bacillus subtilis* DSM 32315 on the pathology, performance, and intestinal microbiome of broiler chickens in a necrotic enteritis challenge. Poult Sci. (2019) 98:3450–63. 10.3382/ps/pey500 30452717 PMC6698186

[133] Stein T. *Bacillus subtilis* antibiotics: structures, syntheses and specific functions. Mol Microbiol. (2005) 56:845–57. 10.1111/j.1365-2958.2005.04587.x 15853875

[134] Piggot PJ Hilbert DW. Sporulation of *Bacillus subtilis*. Curr Opin Microbiol. (2004) 7:579–86. 10.1016/j.mib.2004.10.001 15556029

[135] Schallmey M Singh A Ward OP. Developments in the use of *Bacillus* species for industrial production. Can J Microbiol. (2004) 50:1–17. 10.1139/w03-076 15052317

[136] Bernardeau M Lehtinen MJ Forssten SD Nurminen P. Importance of the gastrointestinal life cycle of *Bacillus* for probiotic functionality. J Food Sci Tech. (2017) 54:2570–84. 10.1007/s13197-017-2688-3 28740315 PMC5502041

[137] Ma M Evaluation of a commercially available probiotic and organic acid blend product on production parameters and economics in broiler breeders. Nutr Food Tech Open Access. (2017) 3:1–5. 10.16966/2470-6086.139

[138] Uyen NQ Hong HA Cutting SM. Enhanced immunization and expression strategies using bacterial spores as heat-stable vaccine delivery vehicles. Vaccine. (2007) 25:356–65. 10.1016/j.vaccine.2006.07.025 16920233

[139] Khalique A Naseem T Haque N Rasool Z Effect of *Bacillus subtilis* Pb6 on growth and gut microflora in *Clostridium perfringens* challenged broilers. Int J Anim Vet Sci. (2017) 10:763–6. 10.5281/zenodo.1340216

[140] Solis-Cruz B Hernandez-Patlan D Petrone VM Pontin KP Latorre JD Beyssac E. Evaluation of a *Bacillus*-based direct-fed microbial on aflatoxin B1 toxic effects, performance, immunologic status, and serum biochemical parameters in broiler chickens. Avian Dis. (2019) 63:659–69. 10.1637/aviandiseases-D-19-00100 31865681

[141] Ruiz L Delgado S Ruas-Madiedo P Sánchez B Margolles A. *Bifidobacteria* and their molecular communication with the immune system. Front Microbiol. (2017) 8:2345. 10.3389/fmicb.2017.02345 29255450 PMC5722804

[142] Ivanov D Emonet C Foata F Affolter M Delley M Fisseha M. A serpin from the gut bacterium bifidobacterium longum inhibits eukaryotic elastase-like serine proteases. J Bio Chem. (2006) 281:17246–52. 10.1074/jbc.M601678200 16627467

[143] Flint HJ Duncan SH Scott KP Louis P. Links between diet, gut microbiota composition and gut metabolism. Proc Nutr Soc. (2015) 74:13–22. 10.1017/S0029665114001463 25268552

[144] Arpaia N Campbell C Fan X Dikiy S van der Veeken J deRoos P. Metabolites produced by commensal bacteria promote peripheral regulatory T-cell generation. Nature. (2013) 504:451–5. 10.1038/nature12726 24226773 PMC3869884

[145] Smith PM Howitt MR Panikov N Michaud M Gallini CA Bohlooly Y. The microbial metabolites, short-chain fatty acids, regulate colonic Treg cell homeostasis. Science. (2013) 341:569–73. 10.1126/science.1241165 23828891 PMC3807819

[146] Schulthess J Pandey S Capitani M Rue-Albrecht KC Arnold I Franchini F. The short chain fatty acid butyrate imprints an antimicrobial program in macrophages. Immunity. (2019) 50:432–45.e7. 10.1016/j.immuni.2018.12.018 30683619 PMC6382411

[147] MéNard O Gafa V Kapel N Rodriguez B Butel MJ Waligora-Dupriet AJ. Characterization of immunostimulatory CpG-rich sequences from different *Bifidobacterium* species. Appl Environ Microbiol. (2010) 76:2846–55. 10.1128/AEM.01714-09 20208019 PMC2863467

[148] Keestra AM de Zoete MR Bouwman LI van Putten JPM. Chicken TLR21 is an innate CpG DNA receptor distinct from mammalian TLR9. J Immunol. (2010) 185:460–7. 10.4049/jimmunol.0901921 20498358

[149] Rajkowska K Kunicka-Styczynska A Phenotypic and genotypic characterization of probiotic yeasts. Biotechnol Biotechnol Equip. (2009) 23:662–5. 10.1080/13102818.2009.10818511

[150] Sen S Mansell TJ. Yeasts as probiotics: mechanisms, outcomes, and future potential. Fungal Gen Bio. (2020) 137:103333. 10.1016/j.fgb.2020.103333 31923554

[151] Schneider SM Girard-Pipau F Filippi J Hebuterne X Moyse D Hinojosa GC. Effects of *Saccharomyces boulardii* on fecal short-chain fatty acids and microflora in patients on long-term total enteral nutrition. World J Gastroenterol. (2005) 11:6165–9. 10.3748/wjg.v11.i39.6165 16273644 PMC4436634

[152] Kiros TG Luise D Derakhshani H Petri R Trevisi P D'Inca R. Effect of live yeast *Saccharomyces cerevisiae* supplementation on the performance and cecum microbial profile of suckling piglets. PLoS ONE. (2019) 14:e0219557. 10.1371/journal.pone.0219557 31329605 PMC6645501

[153] Naimah AK Al-Manhel AJA Al-Shawi MJ Isolation, purification and characterization of antimicrobial peptides produced from *Saccharomyces boulardii*. Int J Peptide Res Therap. (2018) 24:455–61. 10.1007/s10989-017-9632-2

[154] Branco P Francisco D Monteiro M Almeida MG Caldeira J Arneborg N. Antimicrobial properties and death-inducing mechanisms of saccharomycin, a biocide secreted by *Saccharomyces cerevisiae*. Appl Microbiol Biotechnol. (2017) 101:159–71. 10.1007/s00253-016-7755-6 27502415

[155] Reya G Ritun C Kasturi S Inulin induced co-aggregation of *Saccharomyces boulardii* with potential pathogenic bacteria. Int J Probiotics Prebiotics. (2019) 14:18–23. 10.37290/ijpp2641-7197.14:18-23

[156] Negro MJ Ballesteros I Manzanares P Oliva JM Sáez F Ballesteros M. Inulin-containing biomass for ethanol production: carbohydrate extraction and ethanol fermentation. Appl Biochem Biotechnol. (2006) 129–132:922–32. 10.1385/ABAB:132:1:922 16915700

[157] Food and Agriculture Organization (FAO) Technical Meeting on Prebiotics: Food Quality and Standards Service (AGNS) FAO Technical Meeting Report. Rome, FAO (2007). p. 15–16.

[158] Alagawany M Abd El-Hack ME Farag MR Sachan S Karthik K Dhama K. The use of probiotics as eco-friendly alternatives for antibiotics in poultry nutrition. Environ Sci Pollution Res. (2018) 25:10611–8. 10.1007/s11356-018-1687-x 29532377

[159] Markowiak P Slizewska K. The role of probiotics, prebiotics and synbiotics in animal nutrition. Gut Pathog. (2018) 10:21. 10.1186/s13099-018-0250-0 29930711 PMC5989473

[160] Redweik GAJ Stromberg ZR Van Goor A Mellata M. Protection against avian pathogenic *Escherichia coli* and *Salmonella* Kentucky exhibited in chickens given both probiotics and live *Salmonella* vaccine. Poult Sci. (2019) 99:752–62. 10.1016/j.psj.2019.10.038 32029160 PMC7587825

[161] Huys G Vancanneyt M D'Haene K Vankerckhoven V Goossens H Swings J Accuracy of species identity of commercial bacterial cultures intended for probiotic or nutritional use. Res Microbiol. (2006) 157:803–10. 10.1016/j.resmic.2006.06.006 16919915

[162] Tellez G Pixley C Wolfenden RE Layton SL Hargis BM Probiotics/direct fed microbials for *Salmonella* control in poultry. Food Res Int. (2012) 45:628–33. 10.1016/j.foodres.2011.03.047

[163] Seol D Jhang SY Kim H Kim SY Kwak HS Kim SH. Accurate and strict identification of probiotic species based on coverage of whole-metagenome shotgun sequencing data. Front Microbiol. (2019) 10:1683. 10.3389/fmicb.2019.01683 31440213 PMC6693478

[164] Chen T Wu Q Zhou H Deng K Wang X Meng F Assessment of commercial probiotic products in China for labelling accuracy and probiotic characterisation of selected isolates. Int J Dairy Tech. (2017) 70:119–26. 10.1111/1471-0307.12331

[165] Dasti JI Tareen AM Lugert R Zautner AE Gross U. *Campylobacter jejuni*: a brief overview on pathogenicity-associated factors and disease-mediating mechanisms. Int J Med Microbiol. (2010) 300:205–11. 10.1016/j.ijmm.2009.07.002 19665925

[166] European Food Safety Authority (EFSA) Scientific Opinion on Campylobacter in Broiler Meat Production: Control Options and Performance Objectives and/or Targets at Different Stages of the Food Chain. Parma (2011). 10.2903/j.efsa.2011.2105 PMC13129689 42078736

[167] Beery JT Hugdahl MB Doyle MP. Colonization of gastrointestinal tracts of chicks by campylobacter jejuni. Appl Environ Microbiol. (1988) 54:2365–70. 10.1128/AEM.54.10.2365-2370.1988 3060015 PMC204261

[168] Glunder G Spiering N Hinz K (eds.). Investigations on parenteral immunization of chickens with a campylobacter mineral oil vaccine. In: *COST Action 97 Pathogenic Micro-Organisms in Poultry and Eggs*. Budapest (1997).

[169] Ziprin RL Hume ME Young CR Harvey RB. Inoculation of chicks with viable non-colonizing strains of *Campylobacter jejuni*: evaluation of protection against a colonizing strain. Curr Microbiol. (2002) 44:221–3. 10.1007/s00284-001-0088-3 11821932

[170] Noor SM Husband AJ Widders PR. *In ovo* oral vaccination with *Campylobacter jejuni* establishes early development of intestinal immunity in chickens. Br Poult Sci. (1995) 36:563–73. 10.1080/00071669508417802 8590089

[171] Widders PR Thomas LM Long KA Tokhi MA Panaccio M Apos E. The specificity of antibody in chickens immunized to reduce intestinal colonization with *Campylobacter jejuni*. Vet Microbiol. (1998) 64:39–50. 10.1016/S0378-1135(98)00251-X 9874102

[172] Khoury CA Meinersmann RJ. A genetic hybrid of the *Campylobacter jejuni flaA* gene with LT-B of *Escherichia coli* and assessment of the efficacy of the hybrid protein as an oral chicken vaccine. Avian Dis. (1995) 39:812–20. 10.2307/1592418 8719215

[173] Huang JL Yin YX Pan ZM Zhang G Zhu AP Liu XF. Intranasal immunization with chitosan/ pCAGGS-flaA nanoparticles inhibits campylobacter jejuni in a white leghorn model. J Biomed Biotechn. (2010) 2010:589476. 10.1155/2010/589476 20936115 PMC2948919

[174] Neal-McKinney JM Samuelson DR Eucker TP Nissen MS Crespo R Konkel ME Reducing *Campylobacter jejuni* colonization of poultry via vaccination. PLoS ONE. (2014) 9:e114254 10.1371/journal.pone.0114254 25474206 PMC4256221

[175] Annamalai T Pina-Mimbela R Kumar A Binjawadagi B Liu Z Renukaradhya GJ. Evaluation of nanoparticle-encapsulated outer membrane proteins for the control of *Campylobacter jejuni* colonization in chickens. Poult Sci. (2013) 92:2201–11. 10.3382/ps.2012-03004 23873570

[176] Theoret JR Cooper KK Zekarias B Roland KL Law BF Curtiss R. The *Campylobacter jejuni* Dps homologue is important for *in vitro* biofilm formation and cecal colonization of poultry and may serve as a protective antigen for vaccination. Clin Vaccine Immunol. (2012) 19:1426–31. 10.1128/CVI.00151-12 22787197 PMC3428391

[177] Nothaft H Davis B Lock YY Perez-Munoz ME Vinogradov E Walter J. Engineering the *Campylobacter jejuni* N-glycan to create an effective chicken vaccine. Sci Rep. (2016) 6:26511. 10.1038/srep26511 27221144 PMC4879521

[178] Wang C Zhou H Guo F Yang B Su X Lin J. Oral immunization of chickens with *Lactococcus lactis* expressing *cjaA* temporarily reduces *Campylobacter jejuni* colonization. Foodborne Pathog Dis. (2020) 17:366–72. 10.1089/fpd.2019.2727 31718285

[179] Layton SL Morgan MJ Cole K Kwon YM Donoghue DJ Hargis BM. Evaluation of *Salmonella*-vectored *Campylobacter* peptide epitopes for reduction of *Campylobacter jejuni* in broiler chickens. Clin Vaccine Immunol. (2011) 18:449–54. 10.1128/CVI.00379-10 21177910 PMC3067390

[180] Nothaft H Perez-Muñoz ME Gouveia GJ Duar RM Wanford JJ Lango-Scholey L. Coadministration of the *Campylobacter jejuni* N-glycan-based vaccine with probiotics improves vaccine performance in broiler chickens. Appl Environ Microbiol. (2017) 83:e01523–17. 10.1128/AEM.01523-17 28939610 PMC5691412

[181] Sahin O Zhang Q Meitzler JC Harr BS Morishita TY Mohan R. Prevalence, antigenic specificity, and bactericidal activity of poultry anti-*Campylobacter* maternal antibodies. Appl Environ Microbiol. (2001) 67:3951–7. 10.1128/AEM.67.9.3951-3957.2001 11525990 PMC93114

[182] Fernandes da Costa SP Mot D Geeraerts S Bokori-Brown M Van Immerseel F Titball RW. Variable protection against experimental broiler necrotic enteritis after immunization with the C-terminal fragment of *Clostridium perfringens* alpha-toxin and a non-toxic NetB variant. Avian Pathol. (2016) 45:381–8. 10.1080/03079457.2015.1129663 26743457 PMC5044767

[183] Timbermont L Haesebrouck F Ducatelle R Van Immerseel F. Necrotic enteritis in broilers: an updated review on the pathogenesis. Avian Pathol. (2011) 40:341–7. 10.1080/03079457.2011.590967 21812711

[184] Mishra N Smyth JA. Oral vaccination of broiler chickens against necrotic enteritis using a non-virulent NetB positive strain of *Clostridium perfringens* type A. Vaccine. (2017) 35(49 Pt B):6858–65. 10.1016/j.vaccine.2017.10.030 29102330

[185] Jiang Y Mo H Willingham C Wang S Park J-Y Kong W. Protection against necrotic enteritis in broiler chickens by regulated delayed lysis *Salmonella* vaccines. Avian Dis. (2015) 59:475–85. 10.1637/11094-041715-Reg 26629620

[186] Wilde S Jiang Y Tafoya AM Horsman J Yousif M Vazquez LA. *Salmonella*-vectored vaccine delivering three *Clostridium perfringens* antigens protects poultry against necrotic enteritis. PLoS ONE. (2019) 14:e0197721. 10.1371/journal.pone.0197721 30753181 PMC6372158

[187] Licciardi PV Tang ML. Vaccine adjuvant properties of probiotic bacteria. Discov Med. (2011) 12:525–33. 22204769

[188] Ritzi MM Abdelrahman W van-Heerden K Mohnl M Barrett NW Dalloul RA. Combination of probiotics and coccidiosis vaccine enhances protection against an *Eimeria* challenge. Vet Res. (2016) 47:111. 10.1186/s13567-016-0397-y 27825377 PMC5101694

[189] De Vadder F Grasset E Mannerås Holm L Karsenty G Macpherson AJ Olofsson LE. Gut microbiota regulates maturation of the adult enteric nervous system via enteric serotonin networks. Proc Natl Acad Sci USA. (2018) 115:6458–63. 10.1073/pnas.1720017115 29866843 PMC6016808

[190] Dinan TG Stilling RM Stanton C Cryan JF. Collective unconscious: how gut microbes shape human behavior. J Psychiatr Res. (2015) 63:1–9. 10.1016/j.jpsychires.2015.02.021 25772005

[191] Martin CR Osadchiy V Kalani A Mayer EA. The brain-gut-microbiome axis. Cell Mol Gastroenterol Hepatol. (2018) 6:133–48. 10.1016/j.jcmgh.2018.04.003 30023410 PMC6047317

[192] Ball AG Bell EA Sienerth K Uno JK Using a zebrafish model to study the gut microbiota's effect on the brain's neurochemistry and behavior. FASEB. (2018) 32(S1):765.8. 10.1096/fasebj.2018.32.1_supplement.765.8

[193] Lyte M. Probiotics function mechanistically as delivery vehicles for neuroactive compounds: microbial endocrinology in the design and use of probiotics. Bioessays. (2011) 33:574–81. 10.1002/bies.201100024 21732396

[194] Lyte M. Microbial endocrinology: an ongoing personal journey. Adv Exp Med Biol. (2016) 874:1–24. 10.1007/978-3-319-20215-0_1 26589212

[195] Villageliú DN Rasmussen S Lyte M. A microbial endocrinology-based simulated small intestinal medium for the evaluation of neurochemical production by gut microbiota. FEMS Microbiol Ecol. (2018) 94:1–9. 10.1093/femsec/fiy096 29790946

[196] Valcarce DG Martínez-Vázquez JM Riesco MF Robles V. Probiotics reduce anxiety-related behavior in zebrafish. Heliyon. (2020) 6:e03973. 10.1016/j.heliyon.2020.e03973 32435716 PMC7229491

[197] Borrelli L Aceto S Agnisola C De Paolo S Dipineto L Stilling RM. Probiotic modulation of the microbiota-gut-brain axis and behaviour in zebrafish. Sci Rep. (2016) 6:30046. 10.1038/srep30046 27416816 PMC4945902

[198] Calefi AS Fonesca JGS Cohn DWH Honda BTB Costola-de-Souza C Tsugiyama LE. The gut-brain axis interactions during heat stress and avian necrotic enteritis. Poult Sci. (2016) 95:1005–14. 10.3382/ps/pew021 26957631

[199] Kers JG Velkers FC Fischer EAJ Hermes GDA Stegeman JA Smidt H. Host and environmental factors affecting the intestinal microbiota in chickens. Front Microbiol. (2018) 9:235. 10.3389/fmicb.2018.00235 29503637 PMC5820305

